# *UBE3A* deletion enhances the efficiency of immunotherapy in non-small-cell lung cancer

**DOI:** 10.1080/21655979.2022.2069328

**Published:** 2022-05-08

**Authors:** Nan Zhang, Jie Shen, Lanying Gou, Manming Cao, Weimin Ding, Peng Luo, Jian Zhang

**Affiliations:** a Department of Oncology, Zhujiang Hospital, Southern Medical University, Guangzhou, China; b Luoyang Cancer Clinical Diagnosis and treatment Research Center, The Affiliated Luoyang Central Hospital of Zhengzhou University, Luoyang, China

**Keywords:** Immune checkpoint inhibitors, biomarker, non-small-cell lung cancer (NSCLC), *UBE3A*, deletion

## Abstract

Immunotherapy significantly improves the prognosis of advanced lung cancer. It has become an important treatment option for advanced lung cancer. However, there remain many limitations in clinical treatment, and only a small portion of patients can benefit from immunotherapy. Our study aimed to identify markers that can precisely forecast the efficacy of immunotherapy in patients. We analyzed a non-small-cell lung cancer (NSCLC) immune checkpoint inhibitor (ICI) cohort (n=240). We used this discovery cohort to identify CNVs in genes associated with immunotherapy. We further analyzed immune biomarkers and immune infiltration in The Cancer Genome Atlas (TCGA)-NSCLC cohort and the Gene Expression Omnibus (GEO) cohorts. By analyzing an ICI dataset from MSKCC, we found that progression-free survival (PFS) was improved after UBE3A deletion (UBE3A-del). The analysis results showed that UBE3A-del had higher immunocyte infiltration levels and higher expression levels of immune checkpoint biomarkers and affected the enrichment levels of immune signaling pathways. Our results suggest that UBE3A-del can be used as a predictive biomarker of NSCLC to screen for NSCLC patients who may benefit from ICI therapy.

**Abbreviations:** NSCLC: Non-small cell lung cancer; CNV: Copy number variation; ICIs: Immune checkpoint inhibitors; TCGA: The cancer genome atlas; GEO: Gene expression omnibus; GSEA: Gene set enrichment; PFS: Progression-free survival; OS: Overall survival; TMB: Tumor mutational burden; CTLA-4: Cytotoxic T lymphocyte antigen 4; PD-(L)1: Programmed cell death (ligand) 1; MSI: Microsatellite instability; dMMR: DNA mismatch repair; SCNAs: Somatic copy number alterations; TME: Tumor microenvironment; MSK-IMPACT: The Memorial Sloan Kettering-Integrated Mutation Profilng of Actionable; Cancer Targets; FDA: Food and Drug Administration; WES: Whole-exome sequencing; SNP: Single Nucleotide Polymorphisms; FDR: False discovery rate; DCR: Disease control rate; DDR: DNA damage response and repair; MDSCs: Myeloid-derived suppressor cells; FAO: Fatty acid oxidation

## Graphical abstract

**Figure:**
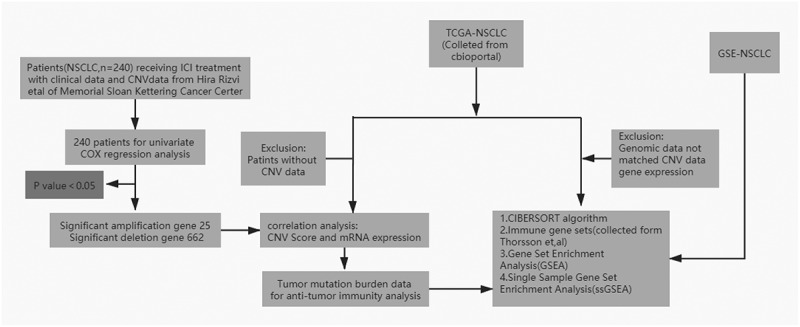

## Highlights

- Our topic is interesting and investigates a current and concrete unmet clinical need, given the lack of reliable predictors of benefit for immunotherapy in NSCLC. We first identified UBE3A-del as a biomarker of immunotherapy response from an ICI-treated cohort(the MSKCC dataset).
- We found that PFS of patients was improved after *UBE3A* deletion in ICI-treated cohort.To investigate the relationship of *UBE3A*-del and immunotherapy, we verified it in the non ICI-treated cohorts, a TCGA dataset and two GEO datasets. After analysis, we determined that *UBE3A*-del has a close relationship to the efficacy of immunotherapy. *UBE3A*-del showed higher TMB in the ICI dataset and TCGA-NSCLC dataset. *UBE3A*-del showed a better disease control rate (DCR).
- Difference analysis of TCGA transcriptome data showed that *UBE3A*-del mRNA expression was lower and that the CNV fraction was positively correlated with RNA expression, *UBE3A*-del methylation degree was higher, expression of some proteins was higher after *UBE3A*-del.
- UBE3A-del enhances immune infiltration. Our results showed that immune genes, immune cells, chemokines and immune checkpoint markers were significantly upregulated in UBE3A-del. Our results show that UBE3A-del enhances antitumour immunity and immunogenicity.
- *UBE3A*-del is enriched in the immune pathway. Our results showed that *UBE3A*-del activated the DNA damage repair mismatch complex pathway and immune pathway.

## Introduction

### Background

The rise of tumor immunotherapy is setting off a new revolution in tumor treatment. Compared with the traditional curative effect, the lasting curative effect is an important advantage of immunotherapy. Immunotherapy has made rapid progress in lung cancer, bringing new hope for advanced lung cancer and becoming an important treatment for advanced lung cancer. However, only a few people show sustained responses, and we need to search for effective biomarkers that can identify potential beneficiaries [1–4].

Previous studies have shown that PD-L1 expression, tumor mutational burden (TMB), microsatellite instability (MSI), deficient DNA mismatch repair (dMMR), immune microenvironment (CD8 + T cell and immune factors), and neoantigen load can predict the response of various tumor types to ICIs [5–13]. However, these predictors have some limitations, and the detection methods and evaluation criteria have not been unified. Therefore, the detection of gene mutations has higher specificity in guiding immunotherapy.

Previous studies have mostly followed the effects of somatic mutations on immunotherapy [14–17]. In the near future, it has been reported that copy number variation is closely related to immunotherapy. Aneuploidy, also known as SCNAs, is considered to be one of the factors leading to tumor development. Teresa Davoli et al. found that the SCNA level could predict markers of cytotoxic immunocyte infiltration better than TMB by exploring a clinical cohort of immunotherapy in patients with melanoma [18,19]. Therefore, SCNA can be used to guide clinical treatment.

**Figure 1.**
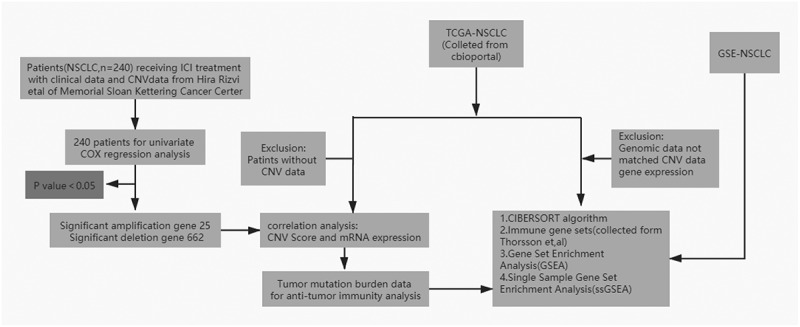
Analysis flow chart.

In this study, CNV and clinical information from multiple databases were collected to identify gene CNVs associated with ICI efficacy. We found that *UBE3A* gene deletion (*UBE3A*-del) showed higher TMB and better PFS, and CNV level of *UBE3A* was correlated with mRNA, methylation and proteomics. After *UBE3A* gene deletion, a better curative effect was achieved by increasing immunocyte infiltration and activating immune pathways [Figure 1].

## Methods

### Clinical cohort

To assess the influence of SCNA on the curative effect of ICIs, we analyzed a discovery cohort. From HIRA Rizvi et al. (https://www.cbioportal.org), a dataset of 240 non-small-cell lung cancer patients was used. MSK-impact sequencing was used in our study, an entire genomic analysis panel authorized for use by the Food and Drug Administration (FDA) [20].

We further analyzed the immune biomarkers and immune infiltration in TCGA-NSCLC, and 586 patients with NSCLC were collected from the TCGA dataset (https://www.cbioportal.org) [21], mainly including whole-exome sequencing (WES), chromosome CNV, transcriptome, methylation, protein chip and clinical data. In addition, Gene Expression Omnibus (GEO) datasets (GSE43580 and GSE63074) containing gene expression data were used to verify the results by analyzing the GEO transcriptome data.

### CNV analysis

We used GISTIC 2.0 to analyze the arm-level SCNA of copy number [22]. For TCGA data, CNVs were measured by the Affymetrix SNP 6.0 chip, the original data were processed by picnic software to obtain the segment file, and usually, the SEG of diploid was zero. Mean values of −0.2 and 0.2 were used to determine whether the region was deleted or amplified. Based on the G score and Q value, CNVs with significant variation were identified. False discovery rate (FDR) values exceeding the level (Q < 0.25) were significant. The results of GISTIC 2.0 were shown by the R package maftools [23].

### Analysis of tumor immunogenicity

The CIBERSORTx deconvolution method was used to estimate the levels of tumor immunocyte infiltration [24]; 22 types of immunocyte infiltration levels in *UBE3A*-del and *UBE3A*-wt were compared, and the outcomes were visualized by the R package ggpubr.

### Pathway enrichment analysis

We used edger to analyze the transcriptome data of the TCGA-NSCLC and GEO cohorts. Then, we used clusterProfiler to analyze the correlation of CNVs and the activation levels of pathways [25,26]. The gene expression of the TCGA-NSCLC and GEO cohorts was analyzed by single-sample gene set enrichment analysis (ssGSEA) with GSVA. Finally, we used the R package pheatmap to visualize the results.

## Statistical analysis

We used the Mann–Whitney U test to analyze TMB discrepancies, mRNA expression, methylation values, immunocyte infiltration levels and immune checkpoint biomarkers from *UBE3A*-del. Kaplan–Meier survival analysis and Cox regression analysis were used to analyze the PFS and OS of *UBE3A*-del and *UBE3A*-wt. We used the R package ggstatplot to estimate the relationship between CNV status and mRNA and methylation in the TCGA dataset. The limma R package was used to analyze the protein chip expression of *UBE3A*-del and *UBE3A*-wt in the TCGA dataset [27]. We used the edgeR package to analyze the differences in immune genes. The significance level was 0.05, and all statistical tests were two-sided. Statistical analysis was completed using R v.3.6.1.

## Results

### UBE3A*-del increases the efficacy of immunotherapy*

**Figure 2.**
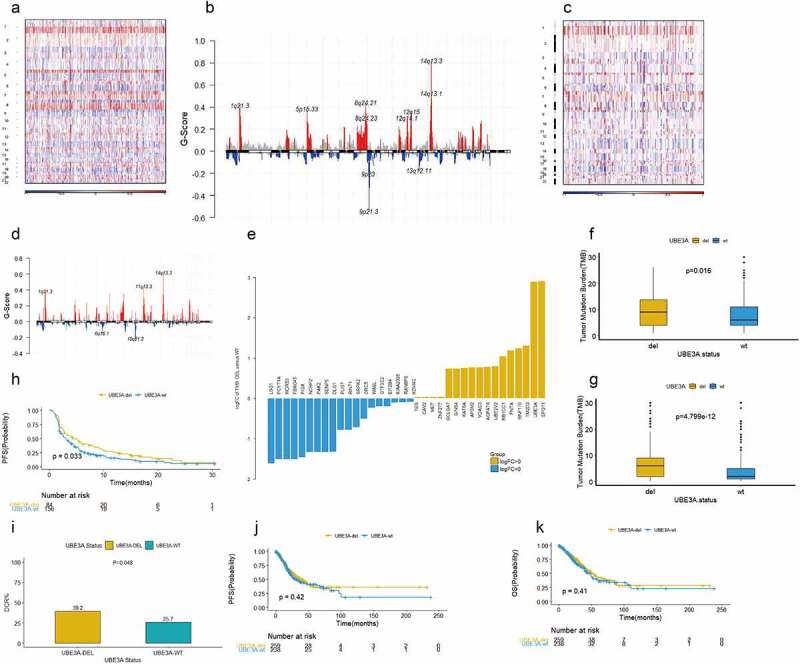
The predictive value of *UBE3A*-del for NSCLC ICI treatment. (a) Results of CNV GISTIC analysis of the TCGA-NSCLC non-ICI cohort:Overview of the chromosomal changes. (b) Comparison of significantly amplified and deleted chromosomes(TCGA). Red represents significantly amplified chromosomes, and blue represents significantly deleted chromosomes. (c) Results of CNV GISTIC analysis of the MSKCC NSCLC ICI cohort:Overview of the chromosomal changes. (d) Comparison of significantly amplified and deleted chromosomes(MSKCC). Red represents significantly amplified chromosomes, and blue represents significantly deleted chromosomes. (e) Relationship between CNV and TMB in the non-ICI cohort of TCGA-NSCLC: Difference between deletion or amplification of the CNV gene and wild-type TMB. (f) Comparison of *UBE3A*-del and *UBE3A*-wt TMB in the NSCLC ICI cohort. (g) Comparison of *UBE3A*-del and *UBE3A*-wt TMB in the NSCLC non-ICI cohort. (h) Survival analysis of PFS in the NSCLC ICI datset between *UBE3A*-del and *UBE3A*-wt. (i) Comparison of the disease control rate (DCR) between *UBE3A*-del and *UBE3A*-wt in the NSCLC ICI cohort. (j) Survival analysis of PFS in the NSCLC non-ICI dataset between *UBE3A*-del and *UBE3A*-wt. (k) Survival analysis of OS in the NSCLC non-ICI dataset between *UBE3A*-del and *UBE3A*-wt.

We performed GISTIC analysis on 586 NSCLC samples from the TCGA cohort (Figure 2(a,b)). The results showed that some chromosomes were significantly amplified (Q < 0.25, Fig. S1A), such as 14q13.3, 1q21.3, 12q15, 5p15.33, and 8q24.21. Some chromosomes were significantly deleted (Q < 0.25, Fig. S1B), such as 9p21.3, 9p23, 13q12.11, and 22q13.32. We verified the results of the ICI cohort according to the GISTIC analysis results(Figure 2(c,d)). GISTIC analysis of 240 NSCLC patients from HIRA Rizvi et al. showed that some chromosomes were significantly amplified (Q < 0.25, Fig S1C), such as 14q13.3, 1q21.3, 7p11.2, 8q24.3, and 5p15.33, and some chromosomes were significantly deleted (Q < 0.25, Fig. S1D). Through the next analysis, genes with significant amplification and significant deletion of chromosomal sites, which were significantly related to SCNA, were found.

We further explored the relationship between CNV and prognosis in 240 NSCLC patients treated with ICIs including available CNV and clinical data from HIRA Rizvi et al. [28]. According to the status of CNVs (deletion or amplification), genes corresponding to significant deletions or amplified chromosomes were included (GISTIC analysis results). Univariate regression analysis was used to analyze the PFS of ICI efficacy. The results showed that 25 genes were significantly amplified (P < 0.05), such as *ABCG1*, and 662 genes were significantly deleted (P < 0.05), such as *UBE3A*. Then, the genes with significant amplification and deletion were verified in the TCGA cohort. According to the CNV amplification or deletion score and RNA expression in the TCGA cohort, a total of 36 genes with correlation coefficients > 0.6 were all significantly deleted genes, such as *UBE3A* and *SPG11*. After analysis, we determined that *UBE3A*-del has a close relationship to the efficacy of immunotherapy. *UBE3A*-del showed higher TMB in the ICI dataset and TCGA-NSCLC dataset (Figure 2(e), p = 0.016, Figure 2(f); p = 2.825e-12, Figure 2(g)).

### UBE3A*-del and clinical outcomes*

In the MSKCC cohort of HIRA Rizvi et al. 84 cases were *UBE3A*-del, and 156 cases were *UBE3A*-wt. All patients were treated with single or combined immune checkpoint inhibitors. Survival analysis showed that *UBE3A*-del prolonged PFS (P = 0.033, Figure 2(h)), and *UBE3A*-del showed a better disease control rate (DCR) (P = 0.048, Figure 2(i)).The expression of PD-L1 was not significantly different between the two groups(P = 0.96,Fig. S1E).

**Figure 3.**
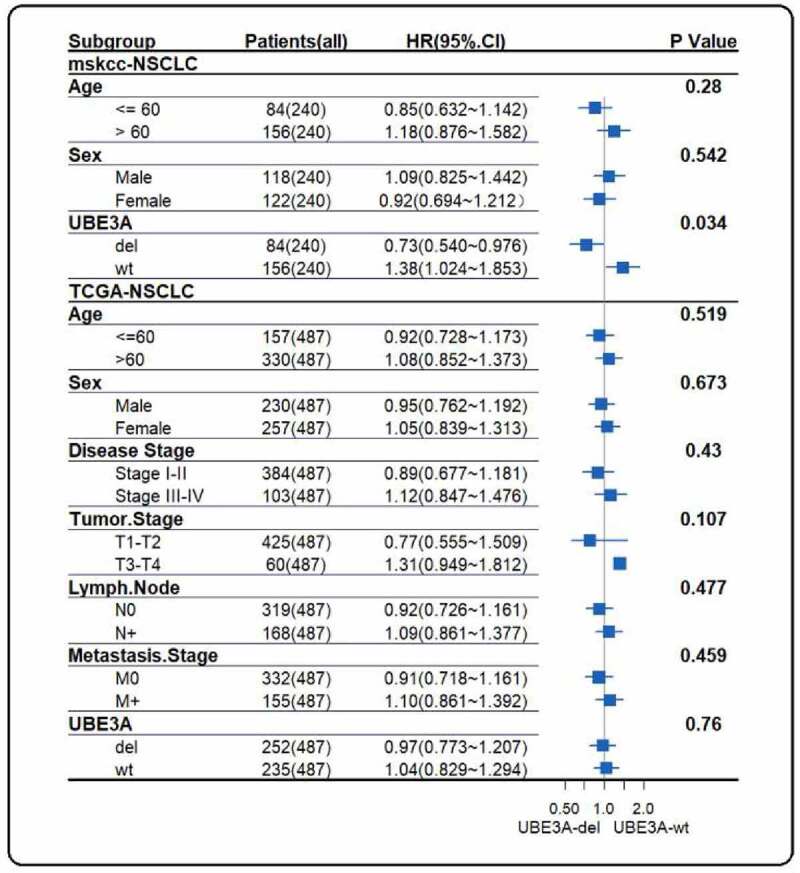
Hierarchical analysis of the MSKCC cohort and TCGA cohort. Age, sex, stage and *UBE3A* CNV status of the MSKCC cohort and TCGA cohort were analyzed.

There were 506 cases of CNV sequencing in the TCGA cohort. According to the status of CNV, 264 cases were *UBE3A*-del, and 242 cases were *UBE3A*-wt. Survival analysis showed that PFS was not significantly different in the TCGA-NSCLC dataset (P = 0.42, Figure 2(j)). Similarly, there was no OS benefit (P = 0.41, Figure 2(k)). Hierarchical analysis of the MSKCC dataset and TCGA dataset was consistent in the age, sex, and stage subgroups (Figure 3, all cases > 0.05). This finding suggests that the improvement in PFS in *UBE3A*-del patients in the MSKCC cohort may be caused by immunotherapy.

### *Correlation between the CNV status of the* UBE3A *gene and RNA, methylation,and protein levels*

**Figure 4.**
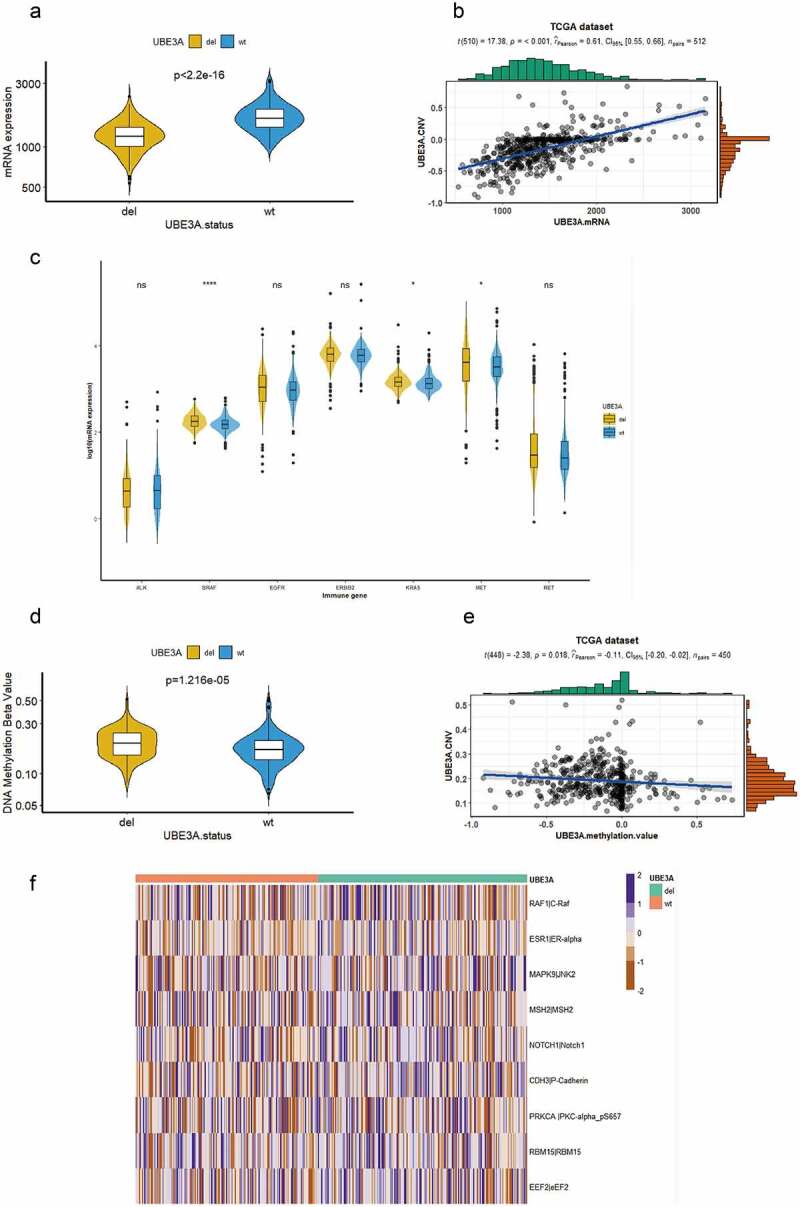
CNV status of the *UBE3A* gene at the RNA, methylation and protein levels and correlation analysis. (a) Difference in the mRNA expression of *UBE3A*-del and *UBE3A*-wt in the TCGA dataset. (b) The relationship between the *UBE3A* CNV fraction and *UBE3A* mRNA expression was analyzed. (c) Differences in common driver genes between *UBE3A*-del and *UBE3A*-wt in the NSCLC dataset. (d) Different degrees of methylation between *UBE3A*-del and *UBE3A*-wt in the NSCLC cohort. (e) The relationship between the *UBE3A* CNV score and the *UBE3A* methylation beta value. (f) Heatmap display of differences in *UBE3A*-del and *UBE3A*-wt protein expression in the NSCLC cohort. Each line represents a protein.

Difference analysis of TCGA transcriptome data showed that *UBE3A*-del mRNA expression was lower (P < 2.2e-16, Figure 4(a)) and that the CNV fraction was positively correlated with RNA expression (P < 0.001, Pearson = 0.61, Figure 4(b)). This finding was consistent with that of a previous report, which was also verified later in the GEO cohort [29]. We analyzed the common driver genes of NSCLC and found that the mRNA expression of *BRAF, KRAS* and *MET* was higher after *UBE3A*-del (P < 2.2e-16, Figure 4(c)), which suggested that *UBE3A*-del patients might be sensitive to targeted therapy, which was in accordance with previous research from Mazieres et al. They revealed that the ICI response rate of lung cancer driven by *KRAS, BRAF* and *MET* was higher than that of other driver genes [30]. The difference analysis of the methylation beta value showed that the *UBE3A*-del methylation degree was higher (P = 1.216e-05, Figure 4(d)), and there was a negative correlation between the two groups (P = 0.018, Pearson = −0.11, Figure 4(e)), which was in accordance with the research of Hyunchul Jung et al. [31]; genomic methylation changes offset the influence of TMB and reduce the effect of immunotherapy.

Finally, by analyzing the protein chip data of TCGA-NSCLC, it was found that the expression of some proteins was higher after *UBE3A*-del (P < 0.05, Figure 4(f)). For example, MAPK and NOTCH1, in our study, the expression of these proteins in *UBE3A*-del exceeded that in *UBE3A*-wt [32,33].

### UBE3A*-del enhances immune infiltration*

**Figure 5.**
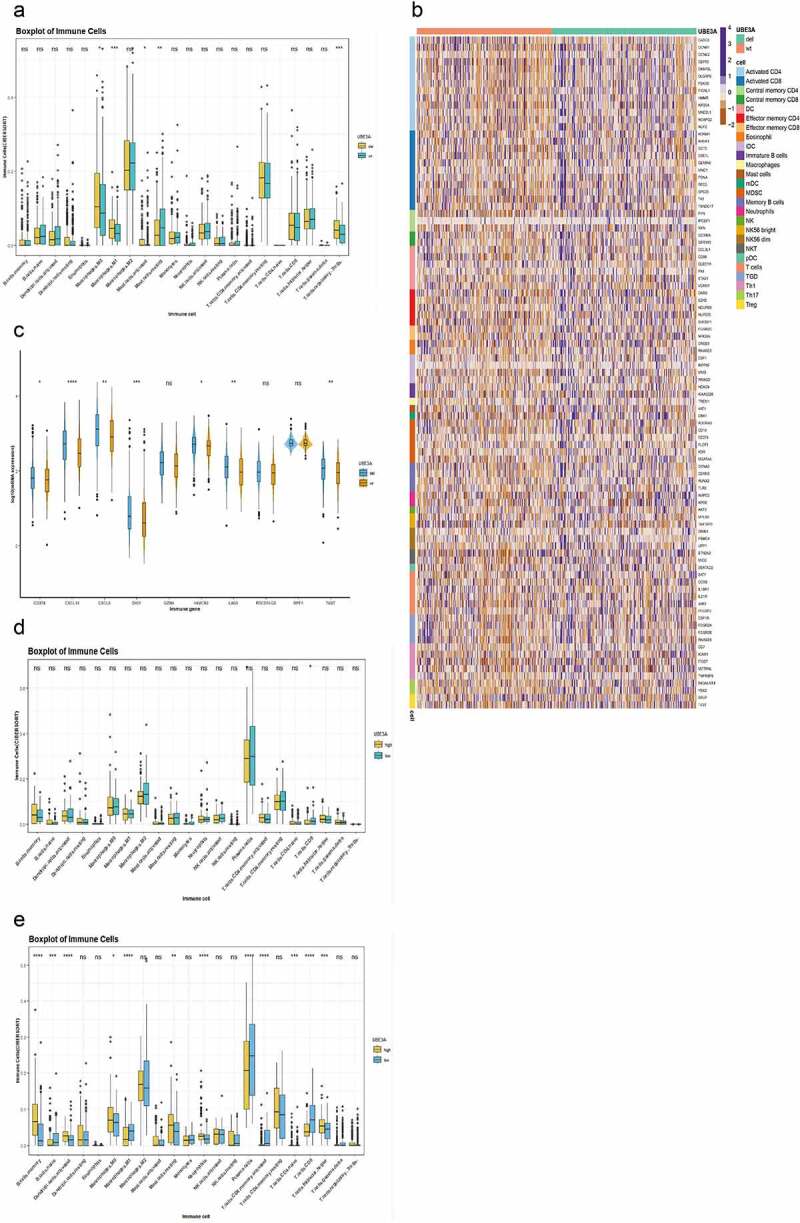
Relationship between *UBE3A*-del and *UBE3A*-wt in immune-infiltrating cell level and immune gene. (a) CIBERSORTx results of the TCGA-NSCLC dataset were based on the differences in the infiltration abundance of 22 kinds of immunocytes with *UBE3A* deletion. (b) Differential expression of immune genes in the TCGA-NSCLC cohort according to *UBE3A*-del and *UBE3A*-wt. Each line represents the expression of one gene. (c) Differential expression of important immune biomarkers, between *UBE3A*-del and *UBE3A*-wt in the TCGA-NSCLC dataset. (d) The results of CIBERSORT analysis of the GSE43580 cohort were analyzed according to the difference in the infiltration abundance of 22 kinds of immunocytes with *UBE3A* deletion. (e) The results of CIBERSORTx of the GSE63074 dataset were analyzed according to the difference in the infiltration abundance of 22 kinds of immunocytes with *UBE3A* deletion.

To research the influence of *UBE3A*-del on immune infiltration, CIBERSORTx was used to analyze the immunocyte infiltration abundance of the TCGA-NSCLC cohort. We discovered that the infiltration degree of M1 macrophages, M0 macrophages, and activated mast cells was increased in *UBE3A*-del (P < 0.05, Figure 5(a)). We further analyzed the immune infiltration of *UBE3A*-del in the TCGA-NSCLC dataset. The results showed that immune genes, such as *AHSA1, ADRM1*, and *CCT5*, which activated CD8 cells, and *CASC5, CCNB1*, and *CCNE2*, which activated CD4 cells, were enriched in *UBE3A*-del (P < 0.05, Figure 5(b))[34]. Moreover, chemokines (*CXCL10* and *CXCL9*) and immune checkpoint markers (*TIGIT, PDCD1* and *LAG3*) were significantly upregulated in *UBE3A*-del (P < 0.05, Figure 5(c)).

**Figure 6.**
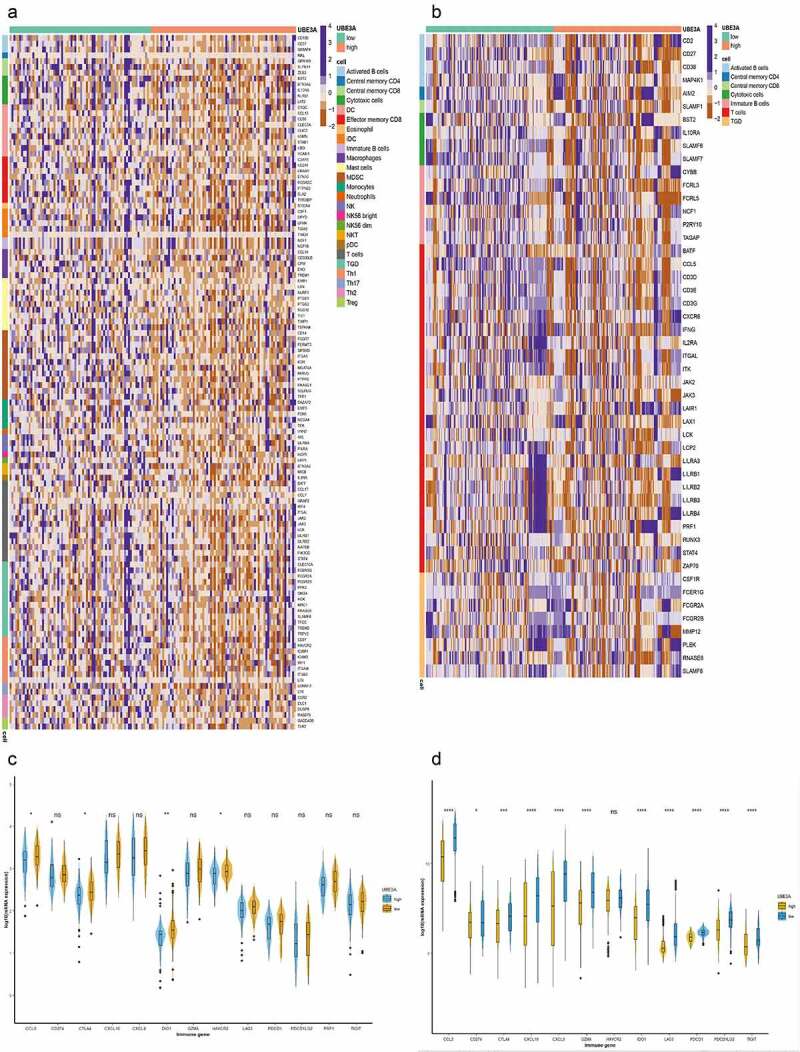
Relationship between *UBE3A*-del and *UBE3A*-wt in immune gene and immune biomarker. (a) Differential expression of immune genes in the GSE43580 dataset according to *UBE3A*-del and *UBE3A*-wt. Each line represents the expression of one gene. (b) Differential expression of immune genes in the GSE63074 dataset according to *UBE3A*-del and *UBE3A*-wt. Each line represents the expression of one gene. (c) Differential expression of important immune genes,between *UBE3A*-del and *UBE3A*-wt in the GSE43580 cohort. (d) Differential expression of important immune genes,between *UBE3A*-del and *UBE3A*-wt in the GSE63074 cohort.

Next, we verified the immune cell infiltration abundance of the GSE43580 and GSE63074 datasets in the GEO cohort by CIBERSORTx. On the basis of the median mRNA expression, we obtained two groups: *UBE3A*-high and *UBE3A*-low. Memory cells, B cells and M1 macrophages were enriched in *UBE3A*-low cells (P < 0.05, Figure 5(d,e)). Some immune-related genes, such as *GPR183, SLFN11, ZEB2, CD2*, and *CD27*, which activate CD8 T cells, and *MAL, CCL5*, and *CXCR6*, which activate CD4 T cells, were significantly upregulated in *UBE3A*-low cells (P < 0.05, Figure 6(a,b)). The expression of cytokines (*CCL5, CXCL9*, and *CXCL10*) and immune checkpoint markers (*CTLA4, DIO1, HAVCR2*, and *PDCD1*) was significantly upregulated in *UBE3A*-low cells (P < 0.05, Figure 6(c,d)). In conclusion, our results show that *UBE3A*-del enhances antitumour immunity and immunogenicity.

### UBE3A*-del is enriched in the immune pathway*

**Figure 7.**
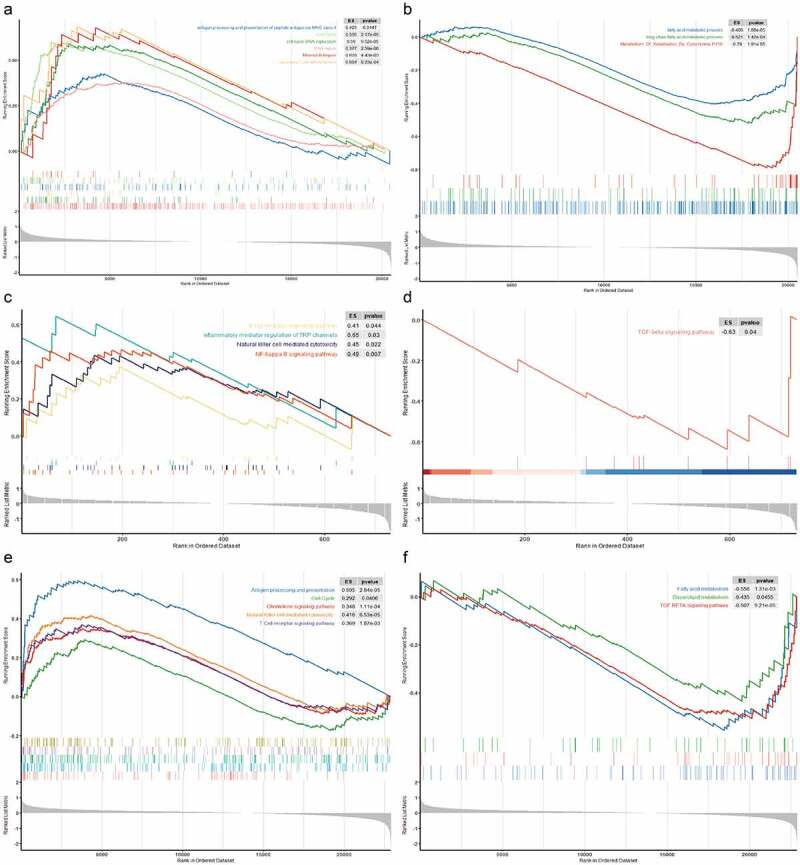
Effect of *UBE3A*-del on the influence of pathways(GSEA). (a) The results of GSEA of the TCGA-NSCLC cohort showed activated pathway in *UBE3A*-del and *UBE3A*-wt pathway enrichment, with p value of 0.05 representing a significant difference. (b) The results of GSEA of the TCGA-NSCLC cohort suggested inhibited pathway in *UBE3A*-del and *UBE3A*-wt pathway enrichment. (c) The results of GSEA of the GSE43580 cohort showed activated pathway in *UBE3A*-low and *UBE3A*-high pathway enrichment, with p value of 0.05 representing a significant difference.The results of GSEA of the GSE43580 cohort showed inhibited pathway in *UBE3A*-low and *UBE3A*-high pathway enrichment. (d) The results of GSEA of the GSE63074 cohort showed activated pathway in *UBE3A*-low and *UBE3A*-high pathway enrichment, with p value of 0.05 representing a significant difference. (e) The results of GSEA of the GSE63074 cohort showed inhibited pathway in *UBE3A*-low and *UBE3A*-high pathway enrichment.

We used the clusterProfiler package to explore the effect of *UBE3A*-del on pathways. Analysis results showed that *UBE3A*-del activated the DNA damage repair mismatch complex pathway. Previous studies have found that DNA damage and DDR gene changes could improve the efficacy of ICIs [35–37]. Activation of the MHC-II pathway enhanced the immune induction of CD4+ lymphocytes (Figure 7(a)). We also found that the downregulation of metabolism-related pathways relieved their immunosuppressive effect [38] (Figure 7(b)). GSEA of the GSE43580 and GSE63074 GEO datasets also showed that *UBE3A*-low activated immune pathways, such as B cell receptors, T cell receptors, antigen presentation, NK cells, cytokines and the cell cycle. In addition, metabolism-related pathways were inhibited after *UBE3A*-del in the TCGA cohort, and fatty acid metabolism-related pathways and TGF-β pathways were inhibited in the *UBE3A*-low GEO cohort (Figure 7(c-f)).

**Figure 8.**
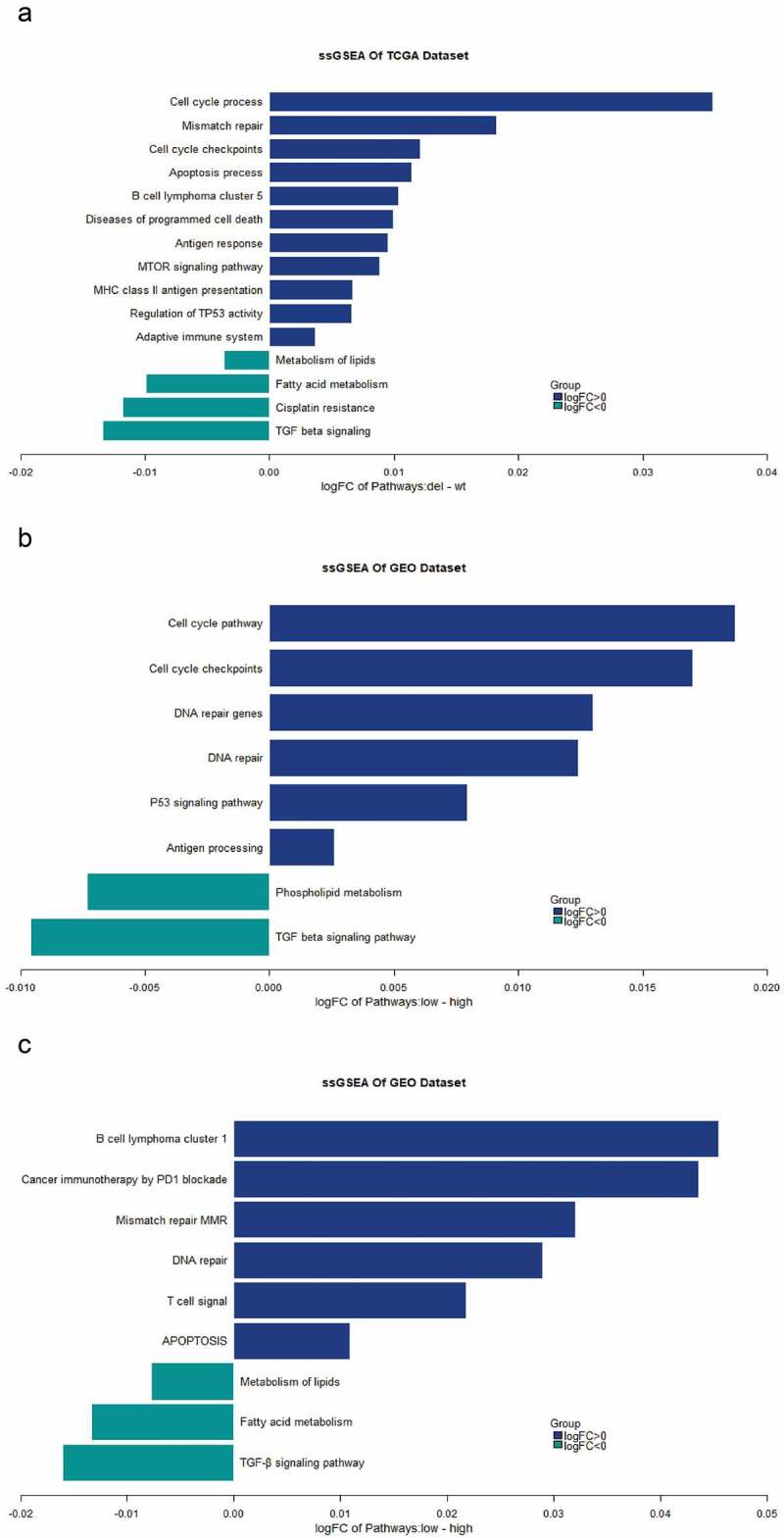
Effect of *UBE3A*-del on the influence of pathways(ssGSEA). (a) TCGA-NSCLC cohort ssGSEA analysis based on *UBE3A*-del and *UBE3A*-wt for the difference in the degree of pathway enrichment. (b) GSE43580 cohort ssGSEA analysis based on *UBE3A*-low and *UBE3A*-high for the difference in the degree of pathway enrichment. (c) GSE63074 cohort ssGSEA analysis based on *UBE3A*-low and *UBE3A*-high for the difference in the degree of pathway enrichment.

To deeply explore the influence of *UBE3A*-del on pathways, we analyzed ssGSEA on the TCGA-NSCLC dataset and GEO dataset. The TCGA analysis results showed that *UBE3A*-del had a significant effect on the activation level of the pathway. The upregulation of autophagy, the cell cycle, B cells, T cells, DNA damage repair, *TP53* and other pathways was significantly higher in *UBE3A*-del than in *UBE3A*-wt (P < 0.05, Figure 8(a)). These pathways had a positive regulatory effect on antitumour immunity. Analysis of the GEO cohort showed that *UBE3A*-low was enriched in the DNA damage repair, cell cycle, antigen presentation and *TP53* pathways, while fatty acid metabolism and TGF-β were inhibited (P < 0.05, Figure 8(b,c)). These results show that UBE3A-del increases the effect of immunotherapy by upregulating the immune pathway.

## Discussion

In this study, we gathered NSCLC datasets, including MSKCC, TCGA and GEO datasets. We selected genes relevant to CNV and prognosis from the MSKCC cohort based on ICI therapy and corresponding clinical data. Finally, we found that UBE3A-del was more relevant to ICIs. *UBE3A*-del was clustered in patients who responded to immunotherapy, which could predict the benefits of patients with *UBE3A*-del.

Although a growing number of studies have demonstrated that chromosome CNV is relevant to ICIs [18,19], there is a deficiency in the accurate relationship between genome changes in CNV and the efficacy of immunotherapy. Romualdo Barroso Sousa et al. found that *PTEN* deletion was reelevated by decreasing tumor immunocyte infiltration and reducing the reaction to immune checkpoint inhibitors [18]. Yuchen et al. suggested that *CCND1* amplification might inhibit the tumor microenvironment (TME) and hinder the immune response of natural hosts, thus affecting the efficacy of ICIs [19]. Our study found that *UBE3A*-del patients had higher TMB and longer PFS related to immunotherapy, and the CNV of *UBE3A* was positively correlated with mRNA expression and negatively correlated with methylation degree, which was consistent with previous research results [26,27,39]. Thijs Stutvoet et al. found that the MAPK pathway was important in inducing PD-L1 expression in lung cancer and might be a potential target for immune checkpoint inhibitors [32]. Hassan Wael et al. revealed that Notch1 could control cell proliferation and apoptosis [33]. In our study, the expression of these proteins in *UBE3A*-del exceeded that in *UBE3A*-wt.

We analyzed the common driver genes of NSCLC and discovered that the mRNA expression of *BRAF, KRAS*, and *MET* was higher after *UBE3A*-del. J Mazieres et al. reported a multicentre study on immunotherapy in 551 NSCLC patients in 2019. The study found that the *KRAS* gene-driven lung cancer ICI treatment response rate was the highest at 26%, followed by *BRAF*, with a response rate of 24%, and the *MET*-driven lung cancer ICI response rate was 16%, which were relatively higher than those of other driver genes [30]. In addition, *UBE3A*-del was highly infiltrated in immunocytes, highly expressed immune genes, and affected immune-, autophagy-, and DNA damage repairable pathways. These sequencing data analyzed based on various databases increased the evidence of the close relationship between *UBE3A*-del and NSCLC ICI efficacy, which supported epigenetics and immunotherapy.

**Figure 9.**
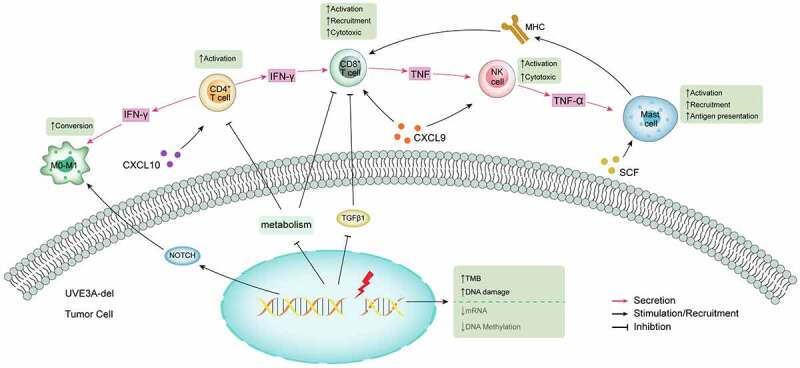
The possible mechanism of improving the prognosis of *UBE3A*-del NSCLC with ICIs.

This is the first report of CNV alteration *UBE3A*-del, which is closely related to NSCLC immunotherapy. *UBE3A* is also known as ubiquitin protein ligase E3A. The ubiquitin proteasome pathway participates in the regulation of the cell cycle, which is the principal protein degradation pathway, signal transduction and DNA damage repair. We summarized the possible mechanism of *UBE3A* in the treatment of ICIs (Figure 9). The deletion of UBE3A leads to gene changes, increased tumor mutation load, aggravated DNA damage, decreased mRNA expression and DNA methylation, thus activating immune-related pathways and consistent metabolism and the TGF-β pathway, which relieves the inhibition of CD4 and CD8 T cells and activates macrophages to activate the body’s response to immunotherapy. Our study revealed that *UBE3A*-del was a potential biomarker of ICIs for NSCLC [40–42].

Previous prediction methods based on markers, including PD-L1 and TMB, are inaccurate, and their shortcomings were not sufficiently accurate. For example, in advanced non-small-cell lung cancer, the PD-L1 detection methods, interpretation criteria, and cutoff values of different immunotherapy drugs are different. Immunotherapy may not be effective for patients with high expression of PD-L1 [43], while it may not be completely ineffective for patients with low PD-L1 expression [44]. Although most studies sustain the high forecast value of TMB [45,46], the detection methods of TMB are distinct, lacking accurate synonymous methods and cutoff values. Therefore, the test of a gene alteration to guide immunotherapy was more accurate, and we could easily obtain the genome change results of patients through sequencing results.

Previous studies have revealed that the Th1 chemokines *CXCL9* and *CXCL10* enhance the tumor attack of effector T cells and increase PD-L1 blockade [47]. Min Yuen Teo et al. found that changes in DDR genes were related to increases in TMB and improvements in clinical prognosis [40,41]. N Auphan et al. suggested that the NF-κB signaling pathway activated cellular immunity by activating immune-related genes [48]. Previous research suggested that TGF-β could form the TME by limiting T cell infiltration, thus inhibiting antitumour immunity [49]. In our study, TGF-β pathways were inhibited in the *UBE3A*-low GEO cohort. In addition, Fokhrul Hossain et al. found that the energy metabolism pathway played an important role in the differentiation and function of immune cells. Myeloid-derived suppressor cells (MDSCs) promote tumor growth by increasing fatty acid uptake and activating fatty acid oxidation (FAO) to inhibit T cell immunity, thus promoting the proliferation and migration of malignant cells. The inhibition of FAO alone could significantly delay the growth of T cell-dependent tumors and increase adoptive T cells and the antitumour effect of treatment [38]. Mauro Di Pilato et al. suggested that a few Treg cells were sufficient to produce this antitumour effect. This result showed that the activation of Treg cells was not enough to fully inhibit the immune system (Figure 5(a)). On the other hand, we might not have enough samples, which led to Treg cells being activated simultaneously with macrophages and mast cells [50]. In our study, *UBE3A*-del resulted in the upregulation of the chemokines *CXCL9* and *CXCL10*, the enrichment of the DDR pathway and the inhibition of the metabolic pathway, which were consistent with the findings of previous reports.

Although UBE3A-del has significant predictive value for NSCLC, the limitation of this study is that we only used bioinformatics to analyze the predictive value of *UBE3A*-del in only the immunotherapy of NSCLC, which is unknown for other tumors, and lacked verification in experiments and clinics. Further validation results are still needed to verify the predictive value of UBE3A-del in the immunotherapy of NSCLC, and it is necessary to expand to other tumors to further analyze its predictive value for immunotherapy.

## Conclusion

Our study provides solid evidence that *UBE3A*-del patients have better PFS with ICIs. Consequently, *UBE3A*-del can be used as a new marker for the ICIs of NSCLC. We still need to verify this result in the clinic and expand the predicted tumor range in clinical practice.

## Supplementary Material

Supplemental Material
