# Supplementary figures and images for: UBE3A deletion enhances the efficiency of immunotherapy in non-small-cell lung cancer
Source: Bioengineered. 2022 May 8;13(5):11577–92. doi: 10.1080/21655979.2022.2069328 (PMC9275990; doi:10.1080/21655979.2022.2069328)

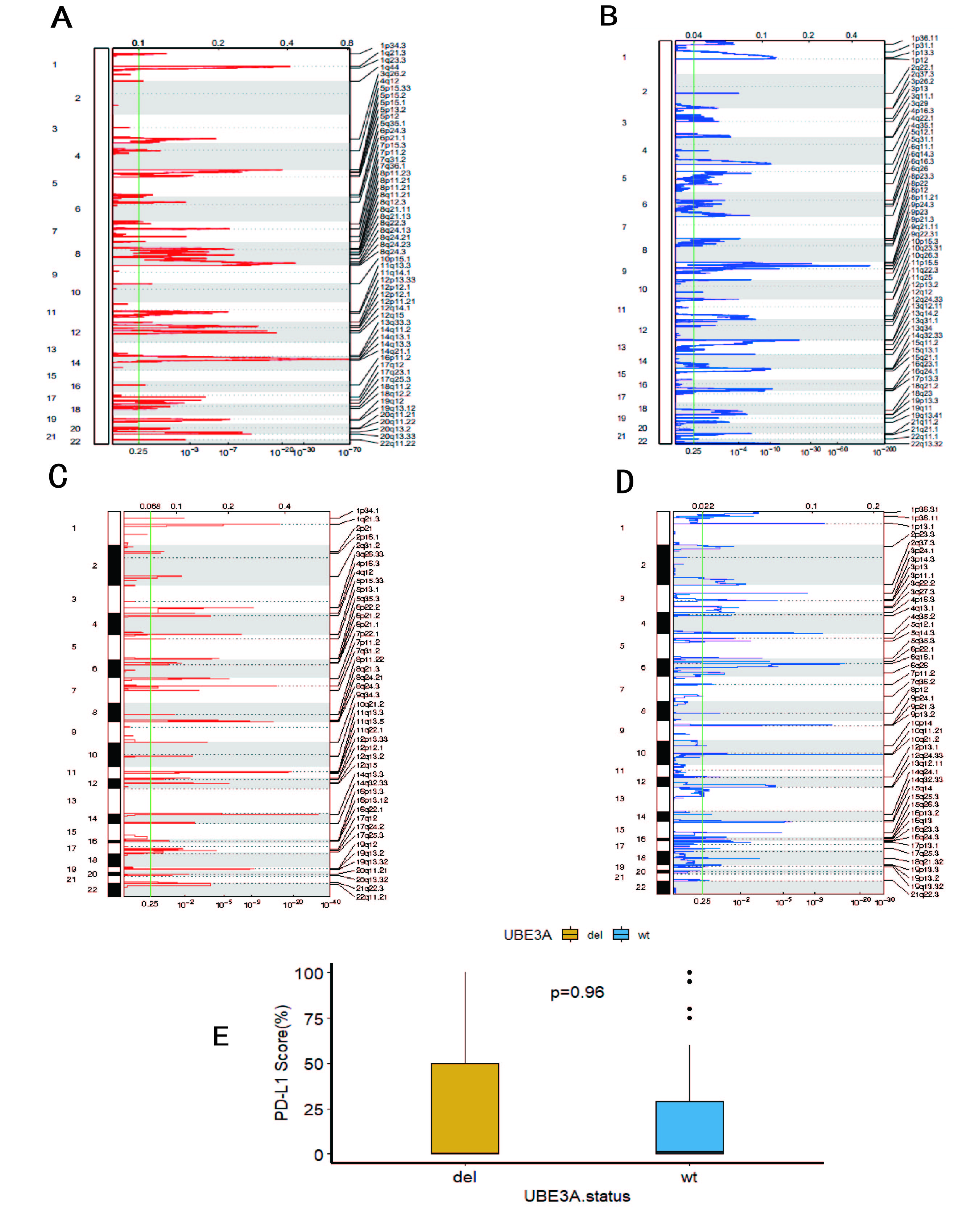

Supplement: Supplemental Material [file KBIE_A_2069328_SM8646.jpg]
